# Bacterial Microbiota Isolated from Cysts of *Globodera rostochiensis* (Nematoda: Heteroderidae)

**DOI:** 10.3390/plants9091146

**Published:** 2020-09-04

**Authors:** Violeta Oro, Magdalena Knezevic, Zoran Dinic, Dusica Delic

**Affiliations:** 1 Department of Plant Diseases, Institute for Plant Protection and Environment, 11000 Belgrade, Serbia; 2 Department of Agrochemistry, Institute of Soil Science, Department of Microbiology, 11000 Belgrade, Serbia; magdalena.knezevic@soilinst.rs (M.K.); zoran.dinic@soilinst.rs (Z.D.); dusica.delic@soilinst.rs (D.D.)

**Keywords:** potato cyst nematodes, Bacilli, Actinobacteria, Alphaproteobacteria, 16S, maximum likelihood, Bayesian inference, climate and edaphic factors

## Abstract

The potato cyst nematode (PCN) *Globodera rostochiensis* is a plant parasite of potato classified into a group of quarantine organisms causing high economic losses worldwide. Due to the long persistence of the parasite in soil, cysts harbor numerous bacteria whose presence can lead to cyst death and population decline. The cysts of *G. rostochiensis* found in two potato fields were used as a source of bacteria. The universal procedure was applied to extract DNA from bacteria which was then sequenced with 16S primers. The aims of the study were to identify bacterial microbiota associated with the PCN populations and to infer their phylogenetic relationships based on the maximum likelihood and Bayesian phylogeny of the 16S sequences. In addition, the impact of the most significant climate and edaphic factors on bacterial diversity were evaluated. Regarding the higher taxonomy, our results indicate that the prevalent bacterial classes were Bacilli, Actinobacteria and Alphaproteobacteria. Phylogenetic analyses clustered *Brevibacterium frigoritolerans* within the family Bacillaceae, confirming its recent reclassification. Long-term climate factors, such as air temperature, insolation hours, humidity and precipitation, as well as the content of soil organic matter, affected the bacterial diversity. The ability of cyst nematodes to persist in soil for a long time qualifies them as a significant natural source to explore the soil bacterial microbiota.

## 1. Introduction

Bacteria are ubiquitous organisms, inhabiting even the most extreme environments like polar snow [1], volcanoes and acidic hot springs [2,3]. The natural soil environment, aside from other microorganisms, harbors as many as 10^6^–10^8^ bacterial cells and 10^6^–10^7^ actinomycete cells per 1 g and around 10^7^ nematodes per 1 m^2^ [4]. 

The potato cyst nematodes (PCNs) *Globodera rostochiensis* and *G. pallida* are plant parasites of potatoes and other Solanaceae plants, classified as quarantine organisms. PCN females are sedentary organisms living inside potato roots with numerous eggs within their enlarged spherical bodies called cysts. The nematodes develop within the eggs to first and second stage juveniles. The latter is the invasive stage, searching for the appropriate host plant. When they find a target host, they start to invade roots, penetrating the host tissue with their stylets and move inside it. Inside the root tissue, they develop into females and males. After mating and fertilization, new eggs and juveniles are produced within the cysts, so the parasitic cycle continues. Some juveniles do not hatch until the following season or favorable conditions, remaining in soil for a long time [5]. The potato cyst nematodes cause up to GBP 300M worth of damage to the potato crop in the EU each year [6].

Both *Globodera* species were brought to Europe with the introduction of potato from South America [7]. Because the PCNs persist in soil, the external and internal areas of cysts harbor numerous microorganisms whose presence can lead to cyst death and population decline, suggesting that they can be potential candidates for use in biocontrol. Microscopic counts using 5-(4,6-dichlorotriazine-2-yl) aminofluorescein staining and in situ hybridization (EUB 338) revealed that cysts contain 2.6 × 10^5^ bacteria [8].

Diverse bacterial species have been reported as nematode antagonists. *Streptomyces avermitilis* and *Pseudomonas fluorescens* were found to possess anthelmintic properties [9]. Nine isolates belonging to *Pseudomonas* and *Streptomyces* species were found to control both fungal pathogens and *Meloidogyne incognita* and were considered as promising biological control agents [10]. Bacterial isolates that inhibited egg hatching of the potato cyst nematodes were mostly from the genus *Bacillus* [11]. Bacterial species of the genus *Pasteuria* were found to be parasites of *Meloidogyne*, *Belonolaimus*, *Pratylenchus*, *Heterodera*, and *Globodera* spp. [12]. The Gram-negative bacterium *Stenotrophomonas (Xanthomonas) maltophilia* G2 was found to have a high nematotoxic activity against the free-living nematode *Panagrellus redivivus*, and the plant parasitic nematode *Bursaphelenchus xylophilus* [13]. *Serratia*, *Curtobacterium*, *Pseudomonas*, *Pantoea*, and *Rhanella* species were nematotoxic toward *B. xylophilus* [14]. Treatment with *B. cereus* strain S2 had a lethal effect on *Caenorhabditis elegans* and *M. incognita* [15].

This study aims to: (i) identify bacterial species associated with two PCN populations, (ii) infer phylogenetic relationships of the bacteria based on the maximum likelihood (ML) and Bayesian inference (BI) of 16S sequences rRNA genes, (iii) evaluate the influence of some microclimate and edaphic factors on bacterial diversity.

## 2. Results and Discussion

**Figure 1:**
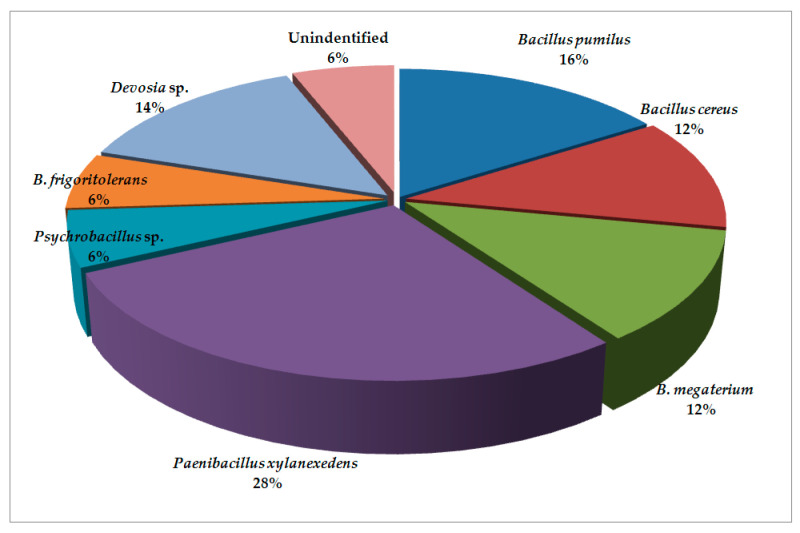
Bacterial microbiota found in cysts from Pozega.

**Figure 2:**
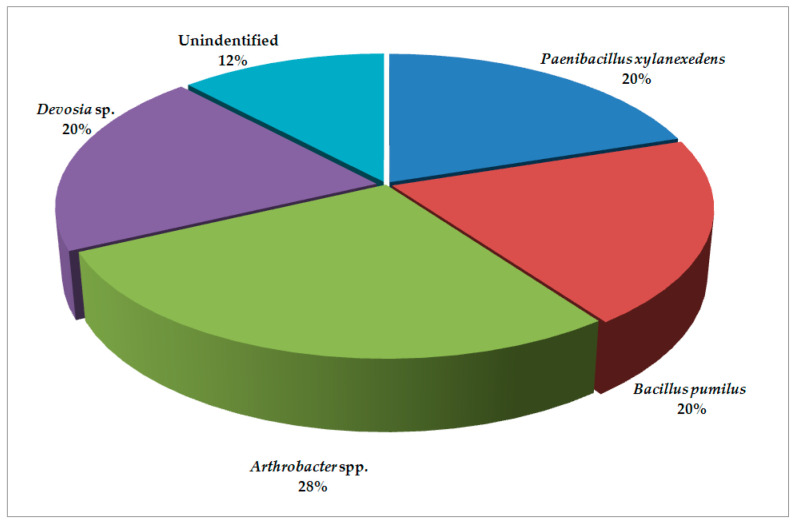
Bacterial microbiota found in cysts from Krupanj.

The results revealed that bacterial microbiota from the locations of Pozega and Krupanj (the Republic of Serbia) generally contain similar species with varying abundance. The cysts obtained from Pozega have more diverse bacterial microbiota (Figure 1) with the presence of 74.0% of members of the class Bacilli and the order Bacillales divided into the families Bacillaceae and Paenibacillaceae. Furthermore, there are 14.0% of members of Proteobacteria, whereas Actinobacteria are present in the lowest percentage (6.0%). The Alphaproteobacteria are represented by the order Rhizobiales and the family Hyphomicrobiaceae (*Devosia* sp.), while Actinobacteria are represented by the order Micrococcales and the family Brevibacteriaceae i.e., *Brevibacterium* sp. The bacterial microbiota of Krupanj (Figure 2) is less diverse, containing the majority of the class Bacilli (40.0%), represented by the families Bacillaceae and Paenibacillaceae as well. The next group is Actinobacteria (28.0%) with the family Micrococcaceae and *Arthrobacter* spp., while the lowest percentage (20.0%) pertains to Alphaproteobacteria represented by the family Hyphomicrobiaceae and *Devosia* sp.

The genus *Bacillus* was the principal genus in Pozega, which was similar to Costa et al. [16] (p. 718), who observed that *Bacillus* was present in 80% of the isolates of the bacterial microbiota of *M. exigua* egg masses in coffee plantations.

*Bacillus* was found not only to be prevalent in the rhizosphere, but also in the phyllosphere. Maximum colonization was shown by the genus *Bacillus* isolated from carrot, cabbage and turnip phyllosphere bacteria [17]. Members of the order Bacillales (*B. pumilus* and *P. xylanexedens*) were found in both locations. In contrast, more Actinobacteria were detected in Krupanj, suggesting that this location was probably more polluted with organic contaminants and the processes of natural bioremediation occurred. In Krupanj, *Arthrobacter* spp. corresponded to 28% of the total bacterial microbiota; likewise, the genus *Arthrobacter* comprised more than 21% of the total soil community of the burned holmoak forest [18].

In comparison with two soil samples from Spain, analyzed by the denaturing gradient gel electrophoresis of bacteria isolated from *M. incognita* and *P. penetrans*, in which the most abundant bacterial classes were Betaproteobacteria, Bacilli and Actinobacteria [19], in our study, the prevalent classes were Bacilli, Actinobacteria and Alphaproteobacteria. The dominance of the order Bacillales was evident in both locations with 80% in Pozega and twice less in Krupanj. In contrast, more Actinobacteria and Alphaproteobacteria (*Arthrobacter* spp. and *Devosia* sp., respectively) were detected in Krupanj.

**Figure 3:**
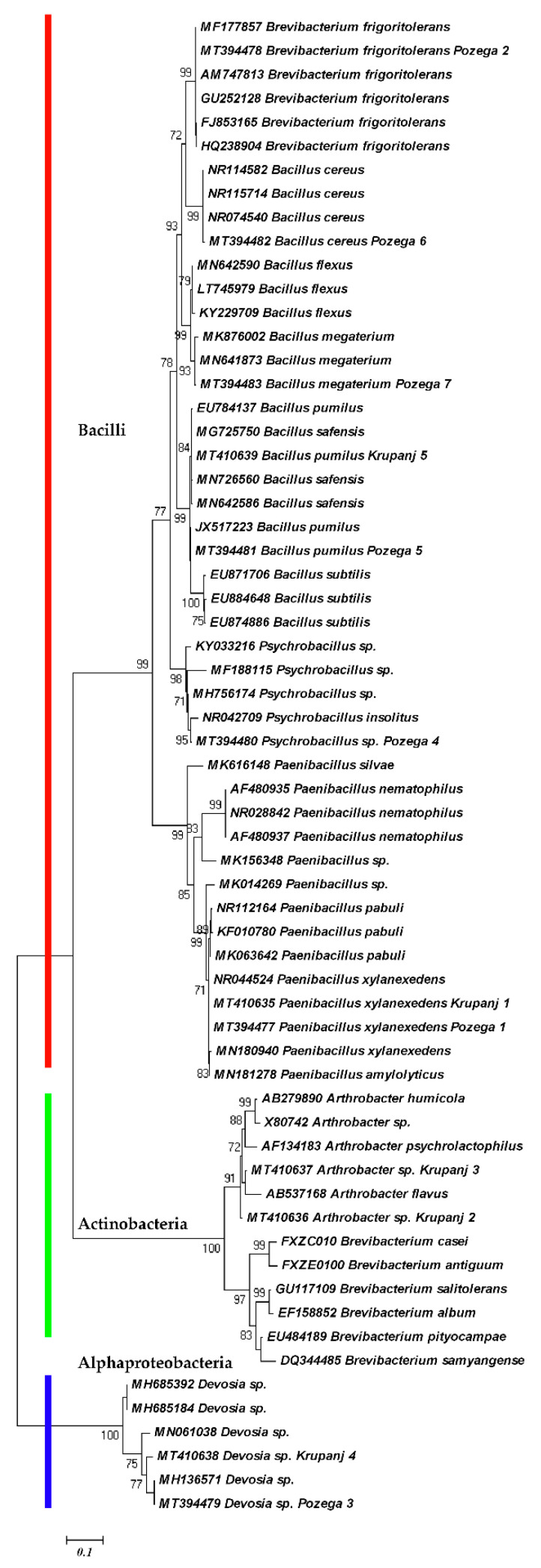
Maximum likelihood phylogenetic tree of bacterial microbiota isolated from *G. rostochiensis* cysts based on 16S sequence region using General Time Reversible (GTR), invariable sites and gamma distribution (GTR + I + G) nucleotide evolution model.

**Figure 4:**
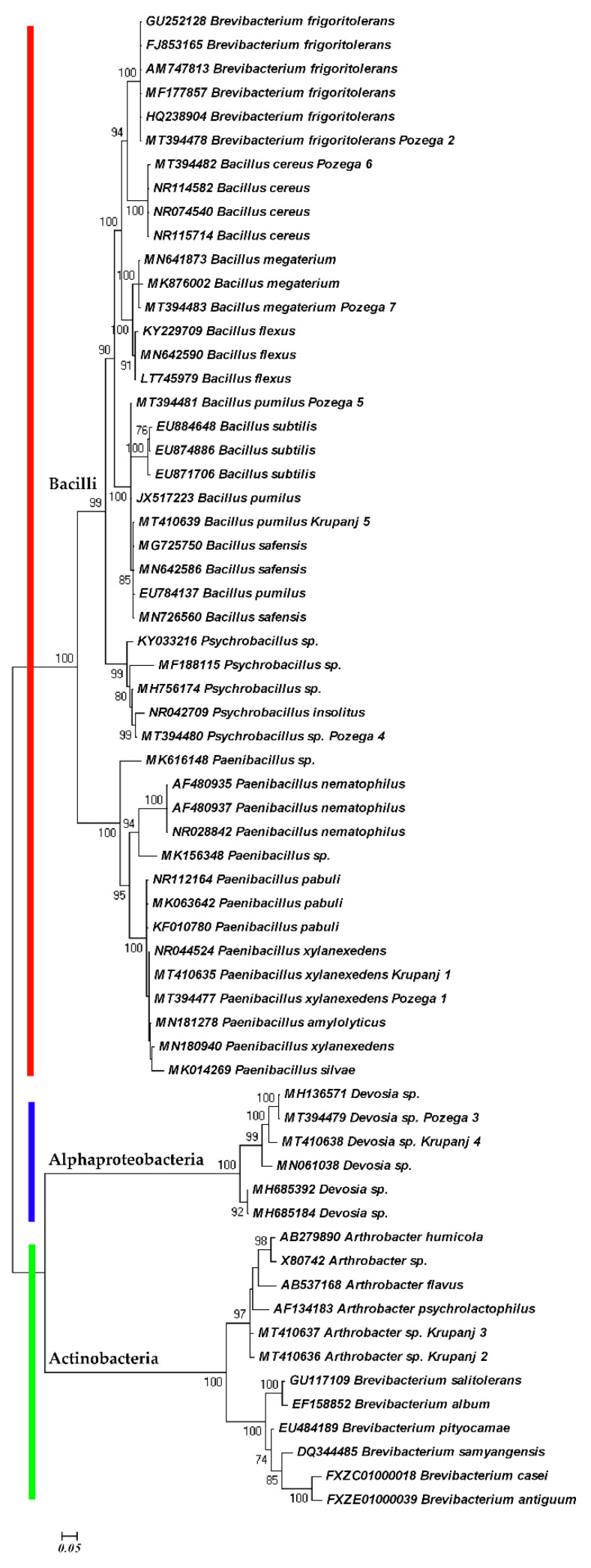
Bayesian phylogenetic tree of bacterial microbiota isolated from *G. rostochiensis* cysts and derived from consensus 50% majority rule based on 16S sequence region using GTR + I + G nucleotide evolution model.

The phylogenetic analyses based on 16S sequences are shown in the Figure 3 and Figure 4. Both ML and BI trees are in agreement and generated three distinct clades. Within the first clade, there are subclades composed of *Bacillus cereus*, *B. megaterium*, *B. flexus*, *B. subtilis*, *B. pumilus* and a *Psychrobacillus* species, representing the family Bacillaceae. The other subclade with *Paenibacillus* spp. represents the family Paenibacillaceae, which, together with the family Bacillaceae, are affiliated to the order Bacillales and the phylum Firmicutes. The difference is that *Devosia* spp. are independent in the ML tree (Figure 3). The *Devosia* species clade represents the family Hyphomicrobiaceae and Alphaproteobacteria linked with the two subclades of Actinobacteria, the subclade of *Arthrobacter* spp. and the subclade of *Brevibacterium* species in the BI tree, because the Bayesian inference considers all the species to be monophyletic (Figure 4). The sequences of *Brevibacterium frigoritolerans* were not clustered with other *Brevibacterium* species. Instead, they were grouped with *Bacillus cereus* species as the closest relatives, suggesting their affiliation to the family Bacillaceae.

Similar observations were reported by other authors. *Brevibacterium frigoritolerans* was in the same group with other *Bacillus* spp., i.e., *B. simplex*, *B. muralis*, *B. psychrosaccharolyticus* [20,21,22]. This bacterium can biosynthesize silver nanoparticles and tolerate silver as some *Bacillus* species can tolerate salt [23]. In addition, *B. frigoritolerans* has the ability to sporulate, thereby providing evidence that this strain is actually a misidentified *Bacillus* sp. [20]. Recently, based on the phenotypic, chemotaxonomic, phylogenetic and genomic characteristics, it has been demonstrated that *B. frigoritolerans* DSM 8801T should belong to the genus *Bacillus*, and to be reclassified as *Bacillus frigoritolerans* [24]. Our study confirms its reclassification and genetic closeness to *B. cereus*. On the other hand, the other species of *Brevibacterium* were clustered together with *Arthrobacter* spp. within Actinobacteria. Apart from *G. rostochiensis*, this species was isolated from juveniles of *B. xylophilus* [25]. Under in vitro bioassay conditions, the isolate of *Brevibacterium frigoritolerans* exhibited bacteremia-like symptoms and induced mortality of the Coleopteran larvae of *Anomala dimidiata* and *Holotrichia longipennis* [26], suggesting its possible use in biocontrol.

Comparisons based on climate factors during the 28-year period (1990–2018) revealed differences between the two locations. Pozega shows the lower values of the air temperatures (optimum, minimum and maximum) and insolation, and the higher values of relative humidity, and cloudiness (Table 1). On the contrary, the values of temperatures, insolation hours and precipitation are higher in Krupanj, whereas the values of relative humidity are lower and there are fewer cloudy days (Table 1).

The honest significant difference (HSD) test demonstrates that there are statistically significant differences between insolation hours and precipitation values. The difference in insolation between locations is almost 500 h with more variation of this factor in Pozega. In contrast, the precipitation sum was higher in Krupanj throughout the year. The insolation itself has a direct impact on the air temperature, making the distinction of this factor between the two locations. A decrease in air temperature causes the decrease in soil temperature, which, in combination with higher relative humidity, favors the environment suitable for cold tolerant species. This fact was confirmed by the presence of *Bacillus frigoritolerans* and a *Psychrobacillus* species in Pozega. Despite the fact that there are no significant differences in air temperature at two locations, the lower annual temperatures in Pozega favored the development of psychrotolerant species. In climate studies, statistical significance does not always provide an adequate basis for decision making; for example, a rise in temperature by two degrees Celsius may not be statistically significant but it can adversely affect the vegetation growth and lead to ecological imbalances [27].

All of the physicochemical properties of the soils, except the content of potassium, were similar in both locations (Table 2). However, the content of soil organic matter in Pozega is higher than in Krupanj, which may explain the more diverse bacterial microbiota in Pozega. Soil with a higher content of organic matter is generally associated with high microbial abundance and diversity [28].

The HSD test demonstrates that there is a significant difference in the amount of potassium between the two locations. Since K^+^ is a major nutritional element for plants, enrichment of K^+^ in the exchange sites due to fertilizer practice can be expected [29], which may indicate high potassium fertilizer inputs in Krupanj.

Regarding the granulometric content of the two examined soils, the smallest clay and silt particles (0.002–0.02 mm) are dominant: 77.7 versus 80.9%. Pozega has a higher content of silt, whereas Krupanj has a higher content of clay. With decreasing particle size, there is an increase in particle number and in the surface area per gram of soil. It is clear that the interfacial area enlarges with an increase in the proportion of the clay–size fraction and, consequently, the opportunities for sorptive interactions between microorganisms and soil particles should increase [30]. The dominance of silt and clay in both soil samples enables good interaction between bacteria and soil.

All found species of the family Bacillaceae have been reported to have high potential as biocontrol agents, which resulted in the development of commercial bionematicidal agents [12]. *Bacillus cereus* strain S2 can produce sphingosine to induce reactive oxygen accumulation, destroy the genital area in nematodes, and inhibit nematode reproduction [15].

*Bacillus pumilus* demonstrated its ability as a potential biocontrol agent against *M. arenaria*, causing 39.8 and 92.8% J2 mortality after three days of exposure to 2.5 and 10% concentrations of bacterial culture, respectively [31]. *Bacillus subtilis* and *B. pumilus* caused the highest reduction (82% and 81.8%, respectively) in *M. incognita* on cowpea [32]. An isolate of *Bacillus megaterium* reduced the root penetration and migration of *M. graminicola* to between 40 and 60% compared with non–treated roots of rice plants [33].

*Paenibacillus nematophilus* has been found to hamper more than 98% of the dispersal of the beneficial nematode *Heterorhabditis megidis* and reduce its infectivity in moth larvae [34].

*Psychrobacillus* species play a role in biodegradation and as antimicrobial agents. *Psychrobacillus soli* could degrade around 72% of oil components at an initial oil concentration of 1500 ppm [35]. Among ten endophytic bacteria, *Psychrobacillus insolitus* and *Curtobacterium oceanosedimentum* showed the highest anticandidal effect against *Candida albicans* and *C. glabrata* [36], while two strains of *P. insolitus* (Mam2 and Ame3) exhibited an inhibitory action against staphylococcal strains isolated from food [37].

*Devosia* and *Arthrobacter* species are best characterized for their bioremediation potential. *Devosia* are well known for their dominance in soil habitats contaminated with various toxins. The uptake and utilization of nutrients for growth and survival was found to be the dominant function of the genus along with the detoxification and degradation of organic pollutants [38].

*Arthrobacter* species were involved in biodegrading a wide variety of compounds, e.g., nicotine, organosilicon compounds, fluorene, the herbicide atrazine [39], and *m*-chlorobenzoate, the central molecule in many pesticides [40]. The majority of the selected strains exhibited a great ability to degrade organic polymers in vitro. Moreover, they possibly present a direct mechanism for plant growth promotion [18]. One of the strains of *A. nicotianae* showed 100% nematicidal activity against *C. elegans* and 91–97% nematicidal activity against *M. incognita* [41].

The higher presence of bioremediators in our samples may indicate the higher presence of pollutants in Krupanj and explain the reduced diversity of bacterial microbiota.

## 3. Materials and Methods

### 3.1. Isolation of Bacteria

The cysts of *G. rostochiensis* found in potato fields near the locations of Pozega (44°04′ N 20°14′ E) and Krupanj (44°18′ N 19°20′ E) were used as a source for screening bacterial microbiota. During the growing season, the soil samples were taken as 50 subsamples/ha in a systematic sampling pattern in order to make approximately one kilogram of composite sample [42].The cyst extraction was done with the Spears apparatus [43] and collected on a 150-µm sieve.

Fifty randomly selected cysts of different ages from each location were surface sterilized with 96% ethanol, 1.5% NaOCl and washed with sterile water according to the procedure applied for *Globodera* juveniles [44]. The cysts were placed on potato dextrose agar (PDA) and maintained for seven days at 25 °C. After the emergence of bacteria on PDA, single bacterial colonies were used to obtain pure cultures by the streakplate method [45].

### 3.2. Molecular Study

The extraction of DNA from bacteria was performed according to a previously described procedure [46]. The PCR reaction mixture consisted of 25 μL 2× PCR Mastermix, 0.5 μL of forward and reverse primers (10 µM), 1 μL of DNA template and PCR-grade water to a total volume of 50 μL. Amplification of the DNA region coding for 16S rRNA was performed by using P0 (5′-GAGAGTTTGATCCTGGCTCAG-3′) and P6 (5′-CTACGGCTACCTTGTTACGA-3′) primers. The temperature profile for the PCR reaction was as follows: 95 °C for 90 s followed by 35 cycles consisting of 95 °C for 30 s, the annealing temperature (60 °C for the first 5 cycles, 55 °C for the next 5 cycles, and 50 °C for the last 25 cycles) for 30 s, and 72 °C for 4 min. The reaction mixture was then incubated at 72 °C for 10 min and at 60 °C for 10 min. The obtained PCR products were purified and sequenced [47]. Phylogenetic analyses were performed with sequences of the isolated bacterial species deposited under accession numbers MT394477-MT394483 (Pozega) and MT410635-MT410639 (Krupanj) and related species from the GenBank nucleotide sequence database, using maximum likelihood (ML) and Bayesian inference (BI) phylogenetic methods. The ML and BI were calculated with the help of PhyML 3.1 [48], and MrBayes 3.1.2 [49] computer programs, respectively. The sequence alignment was done with ClustalW in Mega 4 [50].

The ML tree was obtained with the General Time Reversible model (GTR), invariable sites and gamma distribution (GTR + I + G). The dendrogram obtained by Bayesian inference was created by 2.2 × 10^6^ generations of Markov Chain Monte Carlo, with a sample frequency of 100, and burning function of 20%. The nucleotide evolution model was GTR + I + G as well. Branch supports higher than 70% were shown next to the node.

### 3.3. Statistical Data Analysis

The annual values of climate factors of Pozega and Krupanj were obtained from the official site of the Republic Hydrometeorological Institute of Serbia. The 28-year period (1990–2018) was used for calculating the means of the optimum, maximum, and minimum air temperature, the relative humidity, insolation, cloudiness and precipitation.

The units for the air temperatures were presented in degrees Celsius, the relative air humidity was expressed in percentages, while the duration of the solar radiation (insolation) was expressed in hours. Values of the cloudiness parameter lower than 2 were considered as clear days, while values higher than 6 were considered as cloudy days. The precipitation was expressed in millimeters (Table 1). Soil pH, the content of organic matter, the amount of nitrogen, phosphorus and potassium, as well as the soil granulometric composition (Table 2), were determined according to standard methods and those from the literature [51,52,53,54,55]. The values were compared with a post-ANOVA Tukey’s honest significant difference (HSD) test using DSAASTAT computer program [56], at the 95% confidence interval. Values with the same letter were not significantly different from each other.

## 4. Conclusions

Regarding the higher bacterial taxonomy, our results indicate that the observed locations have similar microbiota, but with a different abundance and species identity. The dominant bacterial phyla are Firmicutes, Actinobacteria and Proteobacteria. Based on 16S sequences, the maximum likelihood and the Bayesian phylogeny clustered the members of the genus *Bacillus, Psychrobacillus* and *Paenibacillus* within the family Bacillaceae. *Brevibacterium frigoritolerans* belonged to the same group with *B. cereus, B. megaterium* and *B. flexus* within the family Bacillaceae, confirming its recent reclassification. Other clades were occupied by *Devosia* and *Arthrobacter* species known for their function in environmental detoxification and the degradation of pesticides. The lower values of air temperatures, insolation, and precipitation and the higher values of relative humidity and cloudiness created conditions for the development of psychrophilic species. The location of Pozega is characterized by psychrotolerant representatives of *Bacillus frigoritolerans,* and a *Psychrobacillus* species. In contrast, Krupanj is characterized by the higher content of potassium, the lower content of organic matter and the presence of bioremediators such as *Devosia* and *Arthrobacter* species. In other words, bacterial species perform as specific indicators of microclimate properties and environmental pollution.

As efforts have been moved towards expanding the source of microorganisms involving the more complex systems in nature [57], nematodes and their related bacterial microbiota present the next biological system to explore the taxonomic diversity of soil bacteria. Nematodes, especially cyst nematodes, are a significant natural source of microorganisms due to their long persistence in soil and the specific environmental conditions inside and outside of the closed area of cysts, in which diverse bacteria are hidden.

## Figures and Tables

**Table 1:** Comparison of annual means and honest significant difference (HSD) of climate factors for two observed locations during the 28-year period (1990–2018).

Climate Factors (Units)	Locations	Means	SD	Range	HSD (*p* = 0.05)
Optimum Air Temperature (°C)	Pozega	10.5	0.6	9.0–10.9	a
	Krupanj	12.6	0.8	10.6–13.4	a
Maximum Air Temperature (°C)	Pozega	17.4	0.7	15.0–18.0	a
	Krupanj	18.6	1.0	15.7–19.7	a
Minimum Air Temperature (°C)	Pozega	5.1	0.7	3.4–6.6	a
	Krupanj	7.7	0.7	5.0–8.8	a
Relative Humidity (%)	Pozega	82.1	3.1	74–85	a
	Krupanj	77.8	4.0	67–86	a
Insolation *(h)	Pozega	1634.6	253.0	1110.5–2064.2	b
	Krupanj	2107.2	188.8	1701.4–2381.7	a
Cloudiness	Pozega	6.5	0.5	5.3–7.1	a
	Krupanj	5.8	0.4	4.7–6.4	a
Precipitation *(mm)	Pozega	762.3	154.4	460.6–1121.5	b
	Krupanj	902.5	160.7	529.2–1242.4	a

* statistically significant.

**Table 2:** Comparison of soil physicochemical parameters and HSD for two observed locations.

Physicochemical Parameters	Locations	Values	HSD (*p* = 0.05)
pH (H_2_O)	Pozega	7.73	a
	Krupanj	7.01	a
pH (1M KCl)	Pozega	6.71	a
	Krupanj	6.26	a
Soil organic matter (%)	Pozega	5.24	a
	Krupanj	3.33	a
N (%)	Pozega	0.22	a
	Krupanj	0.23	a
P_2_O_5_ (mg/100 g)	Pozega	28.21	a
	Krupanj	29.80	a
K_2_O * (mg/100 g)	Pozega	24.50	b
	Krupanj	61.88	a
Sand particles (>0.2 mm)%	Pozega	2.4	a
	Krupanj	1.5	a
Sand particles (0.02–0.2 mm)%	Pozega	19.9	a
	Krupanj	17.6	a
Silt (0.002–0.02 mm)%	Pozega	40.6	a
	Krupanj	35.6	a
Clay (<0.002 mm)%	Pozega	37.1	a
	Krupanj	45.3	a
Silt+Clay (<0.02 mm)%	Pozega	77.7	a
	Krupanj	80.9	a

* statistically significant.
